# Neonatal hypertrophic cardiomyopathy with dyspnoea as the first symptom: a case report

**DOI:** 10.3389/fped.2023.1295539

**Published:** 2023-11-29

**Authors:** Xiaoxia Li, Shu-Jun Hong, Hui Hong, Zhi-Qun Zhang, Jing Li

**Affiliations:** Department of Neonatal Intensive Care Unit, Affiliated to Hangzhou First People’s Hospital, Zhejiang University School of Medicine, Hangzhou, China

**Keywords:** hypertrophic cardiomyopathy, neonate, genetic diagnosis, myosinopathy, case report

## Abstract

Neonatal hypertrophic cardiomyopathy (HCM) is an idiopathic disease characterised by myocardial hypertrophy with normal or small ventricular chambers, a systolic hyperdynamic state and diastolic dysfunction. The etiology, pathogenesis and clinical manifestations of HCM are diverse, and it is likely to progress to sudden cardiac death. The highly heterogeneous nature of this disease determines the difficulty of its diagnosis, and it is especially rare to report that can be diagnosed conclusively in the neonatal period. However, when it does occur, the younger the age of onset is, the higher the mortality rate and the worse the prognosis. The genetic variants and diagnostic timing can affect the life course of the patient. This case report describes a neonate with a family history of HCM who was diagnosed with hypertrophic non-obstructive cardiomyopathy by echocardiography shortly after birth. At 4 years of age, the patient presented with slow weight gain, feeding difficulties, tachypnoea and diaphoresis, and cardiac ultrasound findings suggesting progression to severe hypertrophic obstructive cardiomyopathy, with a high likelihood of arrhythmias, heart failure, pulmonary hypertension, syncope and even sudden death. Neonatal congenital hypertrophic cardiomyopathy is extremely rare and difficult to diagnose before the onset of symptoms. Echocardiography has a definite diagnostic value in hypertrophic cardiomyopathy and helps in early detection and treatment. At the time of clinical diagnosis, children with hypertrophic cardiomyopathy should be asked about their family history and, if necessary, a survey of family members should be conducted for the early detection of mildly ill patients and gene carriers to enable timely intervention and treatment, which remains the focus of our research and efforts.

## Introduction

HCM is a common type of cardiomyopathy in childhood, occurring mostly in boys, and can lead to hypertrophy and fibrosis of the ventricular muscle as well as reduced compliance (1). In the absence of early diagnosis and timely intervention, the disease may cause the patient to develop heart failure, arrhythmias, pulmonary hypertension, syncope and even sudden death. And survivors may even require heart transplantation if they progress to the end stage (2, 3). It is not only one of the most common inherited monogenic heart diseases, but also an important cause of sudden cardiac death in childhood and young adulthood (4). Clinical diagnosis mainly relies on echocardiography and magnetic resonance imaging, combined with clinical manifestations, chest radiographs, electrocardiograms and other special investigations. Even part of the etiological diagnosis may rely on genetic testing. Early and effective diagnosis and treatment can greatly improve the prognosis of children. However, the etiology of HCM in children is complex, the clinical manifestations are highly heterogeneous and heart failure can develop at any age, which makes its diagnosis and treatment more difficult (5, 6). Currently, to the best of our knowledge, there are few case reports of patients with hypertrophic cardiomyopathy diagnosed in the early neonatal period and followed up regularly. In this study, we report a case with a family history of hypertrophic cardiomyopathy and a diagnosis of this type of disease soon after birth, which worsened in spite of regular follow-up and pharmacological treatment. We hope that the findings of this case report will enhance early clinical diagnosis and therapeutic intervention in similar cases in the future. This study is reported in accordance with the CARE guidelines (7).

## Case report 1

**Figure 1:**
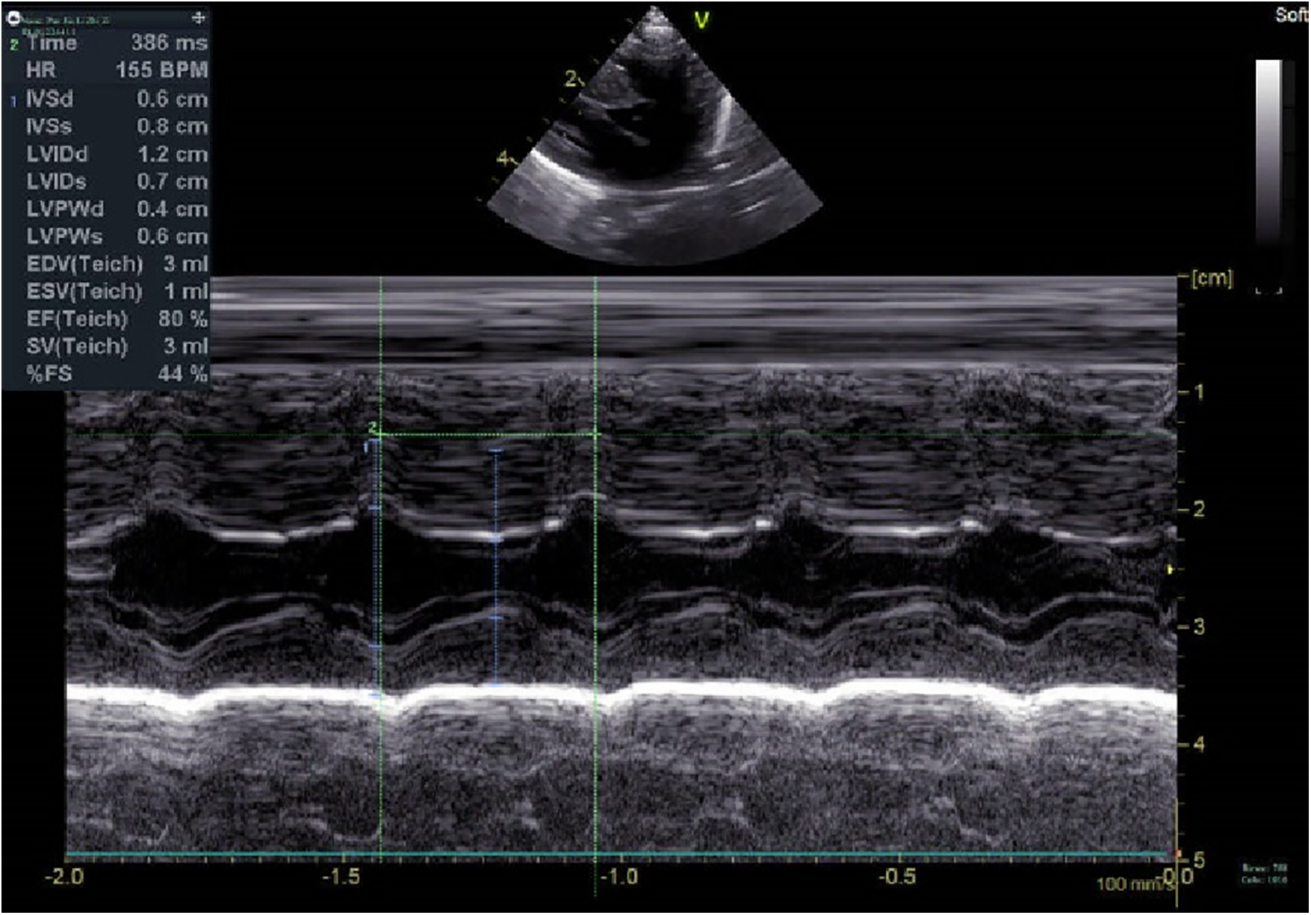
Electrocardiogram at 5 m.

**Figure 2:**
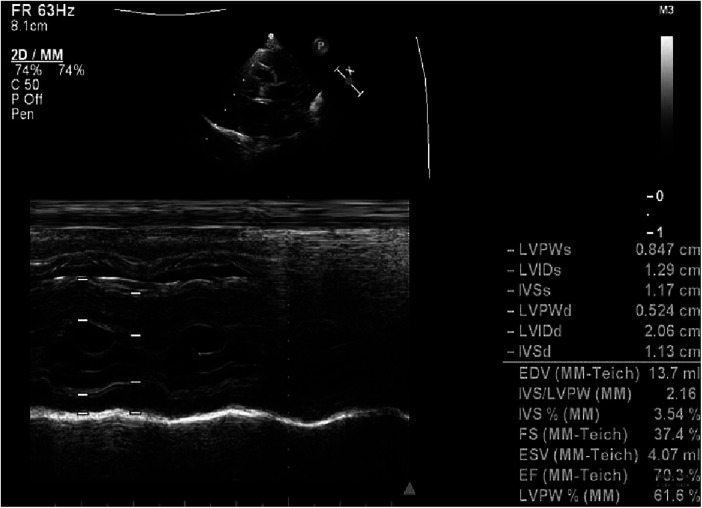
Electrocardiogram at 8 m.

**Figure 3:**
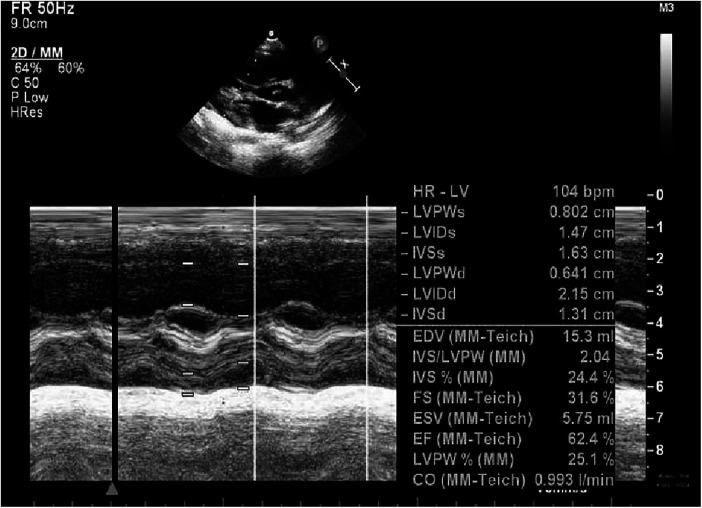
Echocardiography at 1 m.

**Figure 4:**
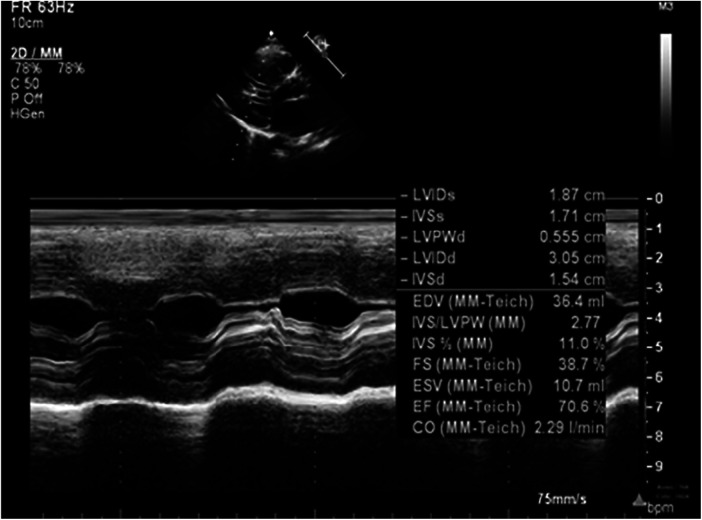
Echocardiography at 4 m.

**Figure 5:**
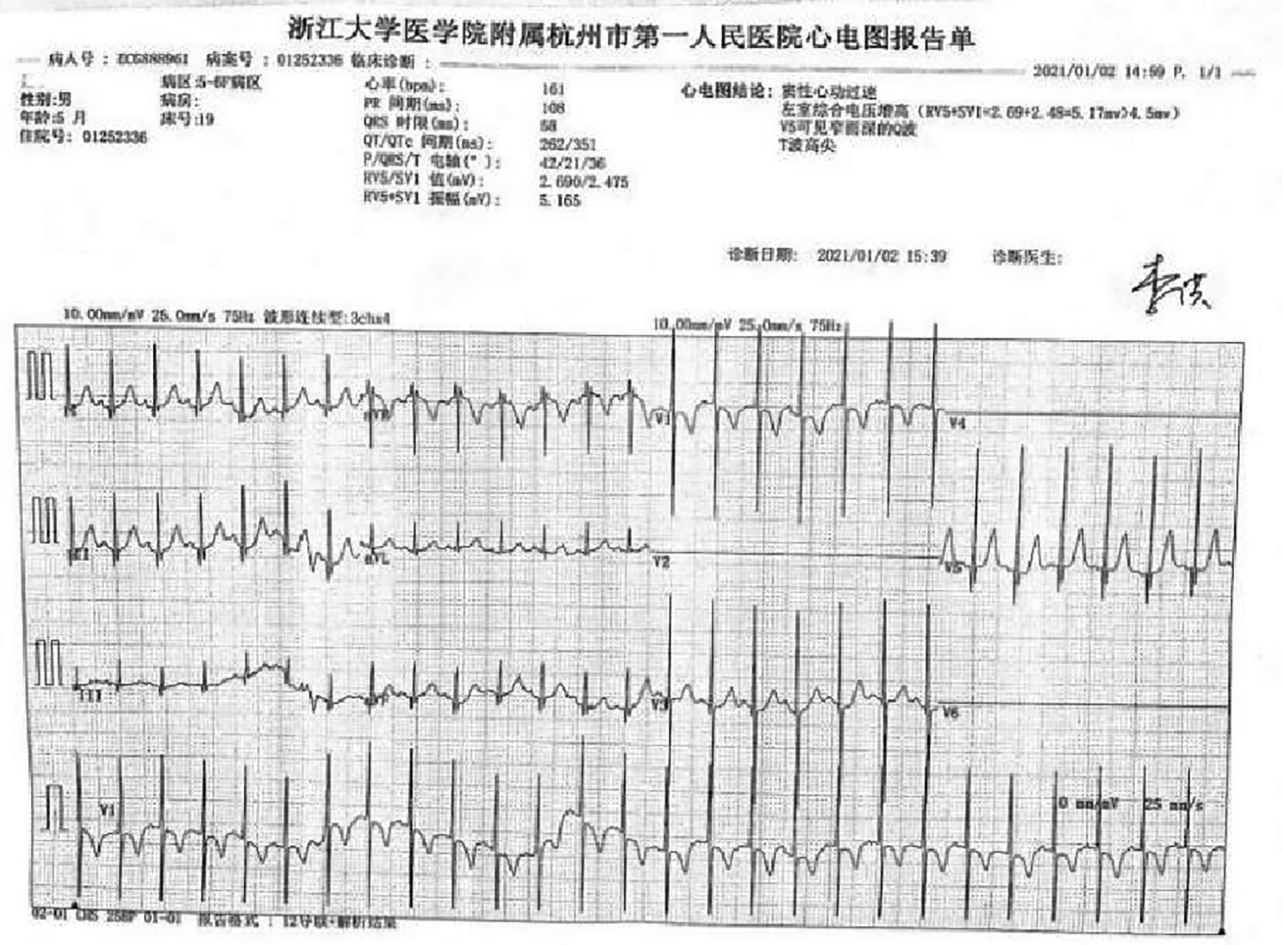
Echocardiography at 12 m.

**Figure 6:**
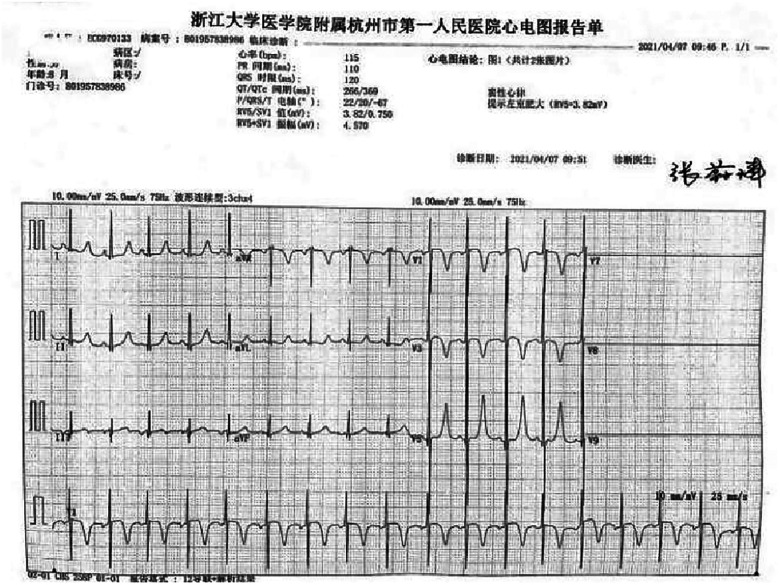
Echocardiography at 32 m.

A preterm baby boy born at 31 weeks of gestational age was admitted to the neonatal intensive care unit (NICU) of Hangzhou First People's Hospital (Hangzhou, China) with dyspnoea and moaning for 19 min after birth. The birth weight was 1,610 g. The child's mother was first diagnosed with hypertrophic non-obstructive cardiomyopathy during an echocardiogram in late pregnancy. And then, she was treated with oral metoprolol. The child has an older sister, now 5 years old, with no history of similar disease. The child's maternal grandfather was diagnosed with the same disease at the age of 50 during a physical examination. The physical examination findings on admission were as follows: temperature 35.7°C, pulse 146 beats/min, respiration 65 breaths/min, blood pressure 52/26 (35) mmHg, transcutaneous oxygen saturation 80%, preterm appearance, weak cry, cyanosis of the mucous membranes of the lips and mouth, weak breath sounds on auscultation of both lungs and no rale heard. The heart rhythm was rhythmic, heart sounds were moderate, and no pathological murmur was heard in the precordial region. Routine blood test results and inflammatory marker levels [including C-reactive protein (CRP) level] were normal on the day of admission, and nucleic acid tests for Streptococcus agalactiae, cytomegalovirus and ureaplasma urealyticum in blood were negative, which together with negative stool, urine, sputum and gastric fluid cultures, ruled out the possibility of intrauterine infection. But the blood gas analysis suggested mild metabolic acidosis. Chest x-ray results showed that the translucency of both lungs was reduced, there was a ground-glass changes, and the size of cardiac shadows was in the normal range. The cardiac ultrasound results showed that the internal diameter of each atrioventricular cavity was normal in size, and a 1.8 mm diagonal fissure was seen in the middle of the interatrial septum. The left ventricular wall was asymmetrically hypertrophied, and the interventricular septum was markedly thickened (6 mm) with enhanced and coarsened echoes. The posterior left ventricular wall was 4 mm thick. No significant obstruction of the left ventricular outflow tract was seen at rest. ECG showed sinus tachycardia. The premature infant was put on nasal continuous positive airway pressure (nCPAP), but the dyspnoea progressively worsened 24 h after birth. When the inhalation oxygen concentration gradually increases to 40%, the symptoms of hypoxia still cannot be improved. Based on the clinical symptoms, signs, auxiliary examinations and treatment, the initial diagnosis of this child: 1. neonatal respiratory distress syndrome with respiratory failure (premature infant), 2. hypertrophic non-obstructive cardiomyopathy. Porcine alveolar surfactant 240 mg was administered by intratracheal drip to improve pulmonary ventilation, and soon the child's dyspnoea was reduced. When the inhalation oxygen concentration was gradually decreased, the transcutaneous oxygen saturation was maintained at more than 90%. At 30d of hospitalisation, the child weighed 2,170 g, already able to receive full intestinal nutrition and out of the ventilator, had no clinical signs of heart failure, arrhythmia, or pulmonary hypertension. Although the child's echocardiogram demonstrated localised myocardial hypertrophy, but he had no signs of obstructive cardiomyopathy at the present time, so the parents were informed of the need for regular follow-up after discharge from the hospital. The follow-up results and treatment process are shown in Table 1. At 3 months of age, the child weighed 3,500 g, had no feeding difficulties and no cyanosis or sweating after crying. A grade 2 systolic murmur could be heard in the 3rd–4th intercostal space at the left edge of the sternum on physical examination. The second follow-up cardiac ultrasound showed asymmetric left ventricular wall hypertrophy (the anterior septum and interventricular septum were up to 8 mm at the thickest point and the posterior left ventricular wall was up to 4 mm in thickness), a rough echogenicity, a disturbed myocardial texture arrangement and hypokinesia. The left ventricular outflow tract did not show any obvious signs of obstruction at rest. The child was given the *β*-blocker metoprolol 3 mg/d(0.8 mg/kg.d) orally twice a day to slow down the process of myocardial hypertrophy. At 5 to 6months of age, the Chest x-ray findings indicated increased thickening of bilateral lung texture and full cardiac shadow. The electrocardiogram results showed sinus tachycardia, increased left ventricular integrated voltage, V5 lead deep q-wave, high wave of sharp T (Figure 1). The 3rd follow-up cardiac ultrasound results showed left atrial enlargement, systole could be seen in the anterior direction of the mitral valve motion (SAM sign means systolic anterior motion), and the posterior leaflet and the anterior leaflet showed motion reversal. The left ventricular wall was asymmetrically hypertrophied and the interventricular septum was markedly thickened. The thickest part was 11 mm and the posterior wall of the left ventricle was 6 mm thick. The dose of metoprolol tablets were increased to 6 mg/d(1.0 mg/kg.d) orally twice a day. Nine months after birth, the child showed pauses in breastfeeding, difficulty in feeding, sweating after activity or crying, no cyanosis and slow increase of body weight. The electrocardiogram was suggestive of sinus tachycardia with increased left ventricular integrated voltage(Figure 2). He weighed only 11 kg at 32 m. From the age of 2 after birth, the dose of metoprolol was increased to 12.5 mg/d(1.2 mg/kg.d). The patient's blood pressure was normal. A grade 3–4 systolic murmur was heard between the mitral valve area and the left sternal border since 4 months after birth. The results of the echocardiogram were reviewed regularly (Figures 3–6): the left atrium was enlarged (anteroposterior and posterior diameters of 15 mm–24 mm, LA/AO 1.33–1.80), the left ventricle and right heart were within the normal range, a bimodal anterior leaflet and a markedly diminished EPSS. The left ventricular wall was asymmetrically hypertrophied (6.0 mm–6.4 mm), the interventricular septum was markedly thickened (11.0 mm–15.4 mm), echogenic enhancement was thickened and protruded into the left ventricular outflow tract, and its motion was diminished. At the age of 4 years, echocardiography showed severe hypertrophic obstructive cardiomyopathy. Anzhen Hospital in Beijing, China, organized Multi-Disciplinary Treatment(MDT) and developed personalized treatment plans. At that time, considering the young age of the patient and the high risk of surgical surgery, after full communication with the patient's parents, a consensus was reached on percutaneous myocardial septal ablation guided by ultrasound. Postoperative cardiac ultrasound showed that the internal diameters of each atrium were normal, and the wall of the left ventricle was thick, with a thickness of 13 mm at the basal segment of the ventricular septum, 11.2 mm in the mid-segment, and 9 mm in the apical segment. The patient did not have any SAM symptoms, and the ventricular wall motion was normal. The anterior mitral valve flowed smoothly without an obvious regurgitation signal. The tricuspid valve was seen to have a trace regurgitation signal. The inner diameter of the left ventricular outflow tract is 13 mm, and the blood flow is smooth. The electrocardiogram result was sinus rhythm. Regular follow-up is still required after surgery. If necessary, this procedure can be repeated.

**Table 1:** Cardiac function classification, electrocardiogram, cardiac ultrasound and pharmacological treatment of HCM case.

Age at birth	<1 m	3 m	4 m	5 m	6 m	9 m	12 m	18 m	24 m	32 m	4 y
Ross score	I	I	II	II	II	III	III	III	IV	IV	III
Heart murmur classification	无	Grade 2	Grade 3–4	Grade 3–4	Grade 3–4	Grade 3–4	Grade 3–4	Grade 3–4	Grade 3–4	Grade 3–4	Grade 2
Interventricular septal thickness (mm)	6	8	11.3	11	11	12	13.1	13	14.6	15.4	9
Left ventricular wall thickness (mm)	4	4	5.2	6	6	6	6.4	6	6.2	5.6	6
Septal/posterior left ventricular wall	1.5	2.0	2.17	1.8	1.8	2.0	2.0	2.2	2.35	2.75	1.5
Left ventricular outflow tract stenosis	No	No	Yes	Yes	Yes	Yes	Yes	Yes	Yes	Yes	No
Left atrial anteroposterior diameter (mm)	11	12	20	15	15	16	22	21	24	24	27
LA/OA	1.43	1.48	1.82	1.33	1.36	1.36	1.80	1.50	1.60	1.50	1.20
SAM sign	−	−	+	+	+	+	+	+	+	+	−
Electrocardiogram results	Sinus tachycardia	Sinus tachycardia	Sinus tachycardia	Sinus tachycardia, Increased left ventricular integrated voltage, V5 lead deep q-wave, high wave of sharp T	Sinus tachycardia, Increased left ventricular integrated voltage	Sinus rhythm, left ventricular hypertrophy	Sinus tachycardia, left ventricular hypertrophy, V5 lead deep q-wave	Sinus rhythm, Increased left ventricular integrated voltage	Sinus bradycardia with a wandering rhythm in the sinus node, Increased left ventricular integrated voltage	Sinus rhythm, Increased left ventricular integrated voltag, high wave of sharp T	Sinus rhythm
Oral Metoprolol	no	3 mg/d(0.8 mg/kg.d)	6 mg/d(1.0 mg/kg.d)	6 mg/d(1.0 mg/kg.d)	6 mg/d(1.0 mg/kg.d)	8 mg/d(1.1 mg/kg.d)	10 mg/d(1.1 mg/kg.d)	10 mg/d(1.1 mg/kg.d)	12.5 mg/d(1.2 mg/kg.d)	12.5 mg/d(1.2 mg/kg.d)	12.5 mg/d(1.2 mg/kg.d)

Genetic generation sequencing was carried out on the child and his parents, maternal grandparents, uncle and cousins(see family tree). A total of four family members, including the child, and a total of three of his relatives, including his mother, maternal grandfather and uncle, were found to have pathogenic variants associated with the clinical phenotype of the subject: the MYBPC3 gene, c.821 + 1G > A, (an ACMG rating of pathogenic, with high pathogenicity, and it is a variant that had been reported previously), and the father, maternal grandmother and three cousins had no variants. The pathogenic locus originated from his mother, and the variant-associated diseases were familial hypertrophic cardiomyopathy type 4, dilated cardiomyopathy type 1MM and left ventricular densification insufficiency type 10, which are pathogenic variants.

## Discussion

Hypertrophic cardiomyopathy (HCM) is a cardiomyopathy characterised by myocardial hypertrophy that is due to a pathogenic variant in the gene encoding myocardial nodule-associated protein or is due to an unknown aetiology, and left ventricular wall involvement is common (8). Hypertrophic cardiomyopathy is rare in children, with an annual incidence of 1.24/100,000 in children under 10 years of age, but has a high mortality rate once diagnosed (9). The clinical manifestations of hypertrophic cardiomyopathy are mostly subclinical, and the first symptom in most patients is sudden cardiac death, which makes it tricky for patients and physicians alike (10). Echocardiography helps in the early detection and treatment of the disease (11). Echocardiography can determine whether there is an obstruction or not based on its LV outflow tract blood flow velocity and pressure step difference and the presence of the SAM phenomenon (12). The clinical presentation in the neonatal period is highly atypical and is commonly seen in cases where the diagnosis of HCM is confirmed only after routine cardiac ultrasound due to other reasons such as prematurity, respiratory distress, and babies younger than the gestational age (13).

In this case,during the first 3 months of life, despite good weight gain and feeding of the child well, cardiac ultrasound found that the interventricular septum was thickened compared with the previous period. In order to slow down the progression of interventricular septal hypertrophy, the child was started on metoprolol (0.8 mg/kg.d) orally. Based on the child's regular outpatient follow-ups on growth, feeding, the cardiac ultrasound and electrocardiogram results, the metoprolol dosage was increased to 1.2 mg/kg.d orally and a diuretic was added to reduce the cardiac load. The child's parents were willing to actively cooperate with the doctor's consultation and treatment. Although the parents of the child were very cooperative throughout the treatment process who played an important role in the improvement of the child's disease, and there were no adverse reactions such as a drop in blood pressure and heart rate during the treatment period, it was regrettable that the treatment plan did not achieve the desired results because the child's cardiac functional status deteriorated with age, and the thickness of the interventricular septum tended to aggravate. This is directly related to the cause of the genetic abnormality. In this case, the low-risk, high-efficacy surgical myocardial resection was able to reverse the progressive heart failure caused by outflow tract obstruction, which is in line with the current recommendations for the treatment of hypertrophic cardiomyopathy (6), and cardiac transplantation is an option for patients with end-stage cardiomyopathy, which greatly improves the outcome.

The aetiology of the disease is still unclear, and Litt MJ pointed out that that there are the following main factors: (1) Genetic factors play a role. Approximately 60% of these patients have a family history, and several siblings of a family may have the disease, which shows a high degree of chromosomal dominant inheritance. Some scholars believe that the DD gene is a risk factor for myocardial hypertrophy and cardiac events in patients with hypertrophic cardiomyopathy. (2) A myocardial hyperresponsive state to circulating catecholamines can play a role. (3) There may be abnormal calcium regulation. It is associated with an increase in intracellular calcium ion concentration (14), thus enabling the effective clinical application of calcium antagonists for the prevention and treatment of the disease (15).

Researches by Marian AJ and Semsarian C revealed that the main causes of HCM are variants in genes encoding myotubular proteins or myotubular-associated structural proteins, which are predominantly inherited in an autosomal dominant manner, with causative or potentially causative genetic variants present in approximately 60% of HCM cases (16, 17). More than 2,000 genetic variants have been confirmed (18). However, the well-documented causative genes are mainly genes encoding myofibrillar proteins, among which the genes encoding β-myosin heavy chain (MYH7) associated with thick myofilaments and cardiac-type myosin binding protein C (MYBPC3) account for a particularly large proportion of patients because they account for approximately 80%-90% of all patients with myofibril variants. In addition, the TNNT2, In reviewTNNI3, MYL2, MYL3, TPM1 and ACTC1 genes are also included, and variants in these genes explain more than 90% of genetically positive HCM, which has been referred to as “myosinopathy” (19, 20). Although HCM is conventionally regarded as a monogenic disease, the final clinical phenotype of HCM is the result of a combination of genotypes, modifying factors, and environmental conditions. The same genetic variant may present different clinical phenotypes depending on the individual's gene expression and epigenetics, as well as differences in genetic background, epigenetic modifications, lifestyle, or other exposure factors (21–23).

In this case, the child was found to have hypertrophic cardiomyopathy on cardiac ultrasound within 1 month after birth. A total of four individuals, including the child himself, his mother, maternal grandfather and uncle, were found to have a pathogenic variant associated with the clinical phenotype of the subject: the MYBPC3 gene, c.821 + 1G > A, (an ACMG rating of pathogenic, with high pathogenicity), the causative locus originating from the mother, and the diseases associated with this variant were familial hypertrophic cardiomyopathy type 4, dilated cardiomyopathy type 1MM, and left ventricular densification insufficiency type 10, a pathogenic variant. The child's disease developed during the neonatal period, and the cardiac ultrasound was suggestive of hypertrophic nonobstructive cardiomyopathy. However, the follow-up showed increasing septal thickness, and the cardiac ultrasound was suggestive of hypertrophic obstructive cardiomyopathy, with a very poor prognosis. It has also been shown that in addition to the classical rare variants that cause disease through a single-gene inheritance pattern, a complex inheritance pattern of common variants may also contribute to HCM, especially in patients who are negative for the known causative genes mentioned above, which should be of concern (19, 20).

In the early 21st century, the prevalence of echocardiographic screening for HCM in China was approximately 80/100,000 in more than 8,000 members of the general population (24). However, the limitations in the means of early screening have led to a likely underestimation of HCM prevalence. With increasing clinical and molecular genetic studies, especially the promotion of family tree screening and the implementation of more sensitive diagnostic cardiac imaging, the prevalence of HCM has been estimated to be at least 1/200 (18).

In our case report, due to timely detection of hypertrophic cardiomyopathy in the neonatal period and the administration of regular cardiac ultrasound, oral medications were added in time to slow the progression of the disease. The importance of early cardiac ultrasonography, genetic testing and screening, and active follow-up management of children with a positive family history who have not yet developed the disease is emphasised. When severe outflow tract obstruction is present, it is important to consult with cardiologists and cardiac surgeons about next steps to improve the patient's ultimate prognosis. Due to the complexity and diversity of the etiology of HCM in children and the difficulty in identifying the etiology, the diagnostic and treatment plan needs to increase the joint decision-making between doctor and patient, the choice of genetic testing and the participation of multidisciplinary physicians in the guidance.

## Data Availability

The datasets used and/or analysed during the current study are available from the corresponding author upon reasonable request.
