# Supplementary figures and images for: Neonatal hypertrophic cardiomyopathy with dyspnoea as the first symptom: a case report
Source: Front Pediatr. 2023 Nov 29;11:1295539. doi: 10.3389/fped.2023.1295539 (PMC10716284; doi:10.3389/fped.2023.1295539)

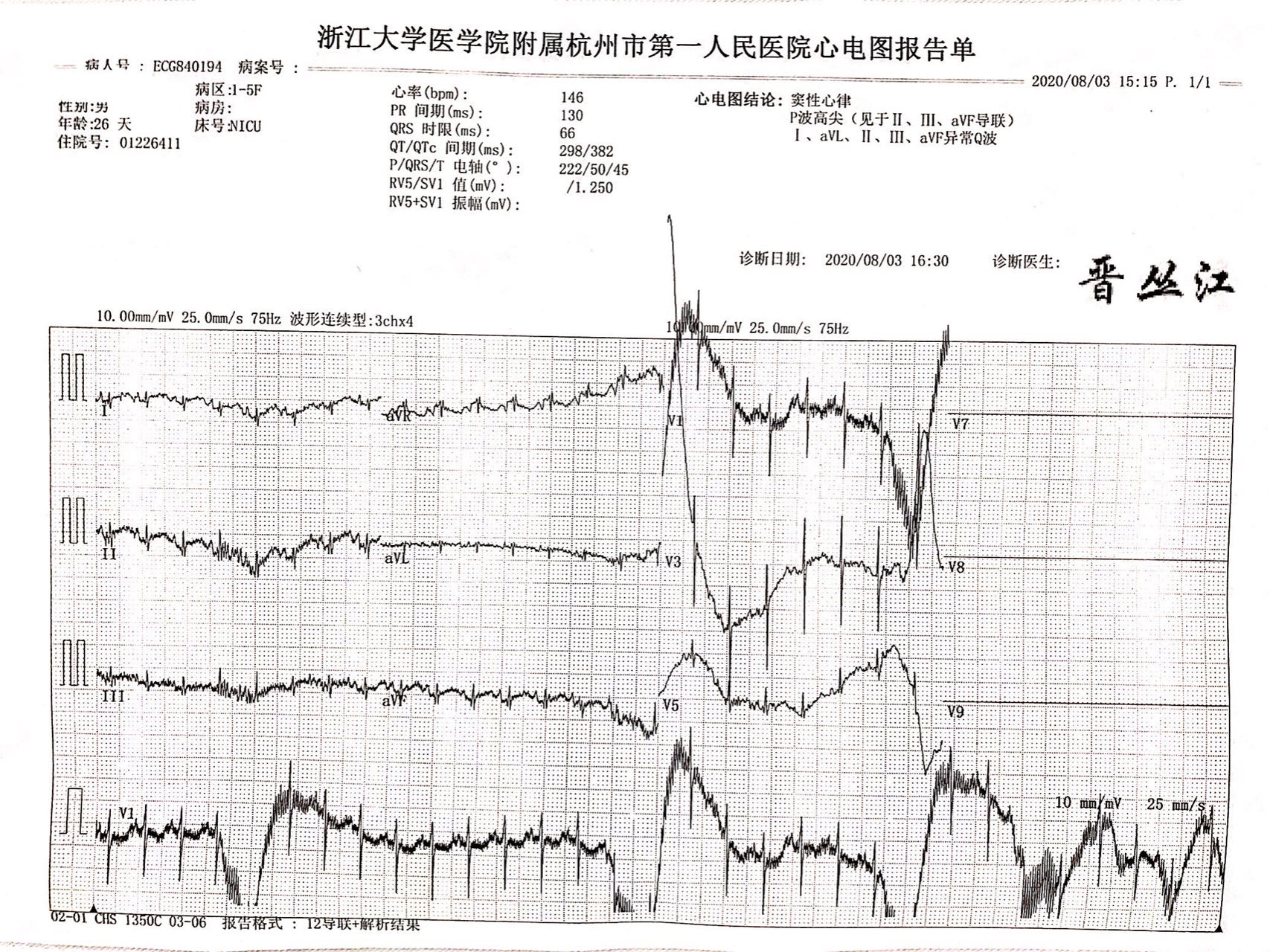

Supplement: Supplementary file 1 [file Image1.jpeg]

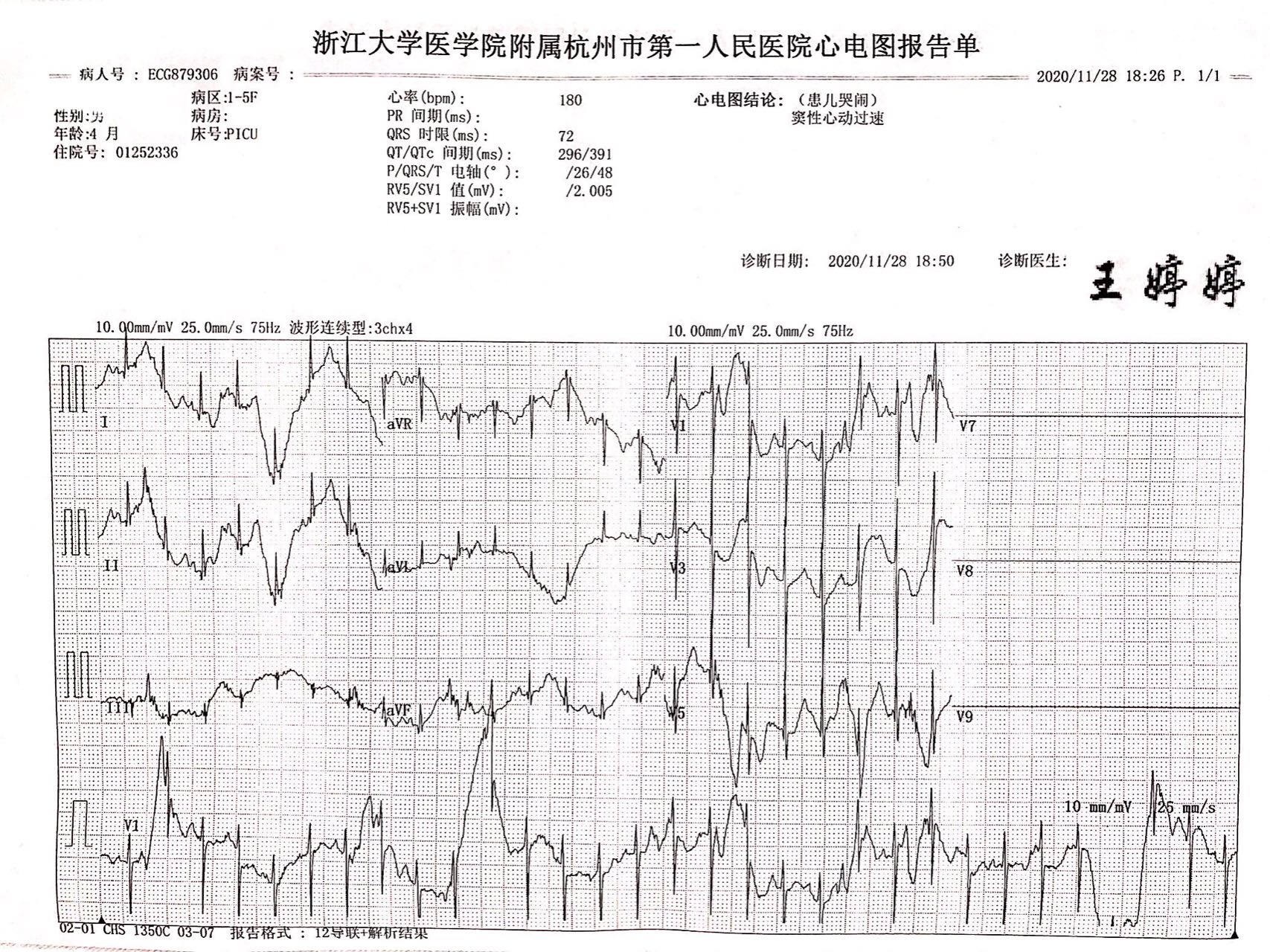

Supplement: Supplementary file 2 [file Image2.jpeg]
